# Young parents’ experiences of pregnancy and parenting during the COVID-19 pandemic: a qualitative study in the United Kingdom

**DOI:** 10.1186/s12889-022-12892-9

**Published:** 2022-03-17

**Authors:** Bettina Moltrecht, Louise J. Dalton, Jeffrey R. Hanna, Clare Law, Elizabeth Rapa

**Affiliations:** 1 Department of Psychiatry, University of Oxford, Warneford Hospital, Oxford, OX3 7JX UK; 2 Evidence-Base Practice Unit, University College London, London, N1 9JH UK; 3 Centre for Longitudinal Studies, University College London, London, WC1H 0NU UK; 4 School of Nursing and Midwifery, Queen’s University Belfast, Belfast, BT9 7BL UK; 5 Centre for Early Child Development, Blackpool Better Start (NSPCC), Blackpool, UK

**Keywords:** Young parents, COVID-19, Perinatal, Remote care, Mental health

## Abstract

Young parents (aged 16–24 years) in the perinatal period are at an increased risk of poor mental health especially during the COVID-19 pandemic, due to multiple risk factors including social and economic instability. COVID-19 related restrictions had profound implications for the delivery of perinatal care services and other support structures for young parents. Investigating young parents’ experiences during the pandemic, including their perceived challenges and needs, is important to inform good practice and provide appropriate support for young parents.

Qualitative interviews were conducted with young parents (*n* = 21) during the COVID-19 pandemic in the United Kingdom from February – May 2021. Data were analysed using thematic analysis.

Three key themes were identified to describe parents’ experiences during the COVID-19 pandemic. Parents reported specific COVID-19 related anxieties and stressors, including worries around contracting the virus and increased feelings of distress due to uncertainty created by the implications of the pandemic. Parents described feeling alone both at home and during antenatal appointments and highlighted the absence of social support as a major area of concern. Parents also felt their perinatal care had been disrupted by the pandemic and experienced difficulties accessing care online or over the phone.

This study highlights the potential impact of the COVID-19 pandemic on young parents, including their mental wellbeing and the perinatal support they were able to access. Insights from this study can inform the support and services offered to families going forward. Specifically, the findings emphasise the importance of (a) supporting both parents during perinatal appointments, (b) providing parents with mental health support early on and (c) finding ways to facilitate communication pathways between professionals and parents.

## Introduction

The impact of the COVID-19 pandemic on the United Kingdom’s (UK) population and in particular their mental health has become a major public health concern [1]. Evidence demonstrates a significant rise in mental health problems across the country during the pandemic [2]. Little research has been conducted which specifically examines the impact of the COVID-19 pandemic on young parents (16–24 years), although this group may have been disproportionally affected by the pandemic, due to the additional social and economic challenges they face [2, 3]. Research suggests that adolescent mothers can be at an increased risk for mental health problems including antenatal and postpartum depression [4]. In addition, some studies have found that their children can be at a higher risk for premature birth and poorer educational achievement [5, 6]. However, there is now increasing evidence that these outcomes may be due in part to prior disadvantage rather than being an adolescent parent. Investigating the experiences of young parents during the pandemic is of importance to inform how support can be provided to them and their children to mitigate the long-term impact of the pandemic.

COVID-19 and related public health restrictions have put a particular strain on parents in the perinatal period [2], as many of them face numerous stressors that are associated with poor mental health including low social support, relationship distress and financial concerns [7, 8]. Young parents (aged 16–24 years) are of particular concern, with a UK-wide survey suggesting that factors including: (a) being a young parent, (b) having little or no income, and (c) not having a partner, were linked to a significantly greater risk of experiencing mental ill-health during the COVID-19 pandemic [2, 9–11].

Studies investigating the experiences of pregnant and new mothers during the pandemic have predominately involved women aged 30 years and older with less research focusing on how young mothers and fathers have navigated this period [12–15]. Evidence has indicated greater levels of psychological distress, anxiety and depression in perinatal women [10, 15, 16], which is of concern, as poor maternal mental health has been shown to be associated with adverse child outcomes including an increased risk of mental and physical health difficulties [17].

COVID-19 restrictions in the UK have had specific implications for fathers with many being unable to attend maternity appointments or be present at the delivery of their baby [18]. Despite policy changes before the pandemic recognising the needs of fathers and the importance of improving paternal support [19], restrictions to services during the pandemic have left many fathers and families without psychosocial support [20, 21]. Men are also at elevated risk of mental health difficulties during the transition to parenthood [22, 23], and these have also been shown to negatively impact children’s development [24, 25]. One study involving young Hispanic fathers in the United States during the pandemic reported that fathers had significant worries about societal expectations to provide for their family, as well as concerns related to job insecurity and future provisions for their family [21].

Research exploring the experiences of young mothers and fathers during the COVID-19 pandemic will aid our understanding of the challenges and needs of this specific group. The resulting insights may have important practical implications in terms of the provision of support needed to mitigate the anticipated inter-generational impact of the pandemic on parents and their children.

### Aims and objectives

The aim of this study is to explore young parents’ experiences and perceptions of becoming and being parents during the COVID-19 pandemic in the United Kingdom. The objectives are to investigate:

1. how young parents navigated the perinatal and postnatal periods during the COVID-19 pandemic, and
2. young parents’ perceptions of whether (and if so, how) they could be better supported during the pandemic and in the future.

## Methods

This is an exploratory qualitative study using semi-structured in-depth interviews. The study is reported following the Standards for Reporting Qualitative Research framework (SRQR [26]).

### Participants

Using convenience and volunteer sampling 21 parents were recruited between February and May 2021. Participants were eligible if they were between the ages of 16 and 24 years, had either been expecting a baby during the COVID-19 pandemic or had a child on or after March 2019, and resided in the UK. Due to the challenges of recruiting young fathers to the study, the research team made the decision to include one father aged 27 years, to ensure fathers’ voices were heard in this study.

### Study recruitment

Participants were invited to take part in a one-off interview by responding to a poster advert. The poster was developed by the research team. The poster was shared via Facebook and Twitter by team members and through relevant professional networks and organisations that have an interest in, or provide parental mental health support in the UK, such as perinatal mental health teams, health visitor services, midwifery networks, and community groups.

The poster included a link to an online information sheet containing details about the study and an online expression of interest form. Interested individuals were contacted via email to assess their eligibility and agree on a time for the interview. Individuals provided informed and written consent prior to taking part in the interview.

### Data collection

Interviews were conducted by three researchers [BM, JRH, LJD], with the majority being conducted by the first author [BM]. A semi-structured interview guide was developed, informed by the literature, study aims and the research team’s expertise (See Table 1). The guide was iteratively reviewed throughout the study. Interviews were conducted on the telephone, audio recorded, and later transcribed by an external provider. Interviews lasted between 20 and 72 min (mean average = 48 min). Data collection was terminated once no further categories were identified. All participants received a £25 voucher for taking part.

**Table 1:** Semi-structured topic guide for interviews

Initial topics based on literature and study aims
•Young parents’ experiences during COVID-19 pandemic ○ Including pregnancy, giving birth and postpartum
•Young parents’ perceptions of their health, social and emotional needs during COVID-19 pandemic
•Young parents’ perceptions of support and ways to cope during COVID-19 pandemic
Additional topics specifically addressed in interviews
•Young parents’ use of online and digital resources
•Impact of COVID-19 pandemic on young parents’ mental health

### Data analysis

Data were analysed using an inductive coding approach following the principles of reflexive thematic analysis [27–29]. Initially the first author [BM] read and re-read all of the transcripts while taking reflective notes. Line-by-line coding of the first five transcripts was conducted to create an initial list of codes, which were reviewed and refined with the research team through critical dialogue. All transcripts were imported to NVivo Pro V12 and codes were created to systemically capture interesting aspects of the data across the entire dataset. The initial list of codes was further extended and refined throughout the entire coding process. To promote study rigour and maintain a connection to the project, eight transcripts were second coded by the three co-authors [JRH, LJD, ER]. Themes were identified based on the existing coding and organised into themes with the help of mind-mapping activities [29]. The final set of themes was refined through discussions with the research team. This led to one main theme being reorganised into two of the existing subthemes.

### Reflexivity, research group and context

The lead author (BM) holds a critical realist approach, which suggests that the present findings do not allow us to objectively, and fully access reality and that the picture presented here is based on participants’ subjective experiences and is further influenced by the researchers who analysed and interpreted the data. The research team consists of four female and one male researcher, all of White ethnicity and at different career and life stages.

Two authors (BM and LJD) have extensive clinical experiences working with parents and young children. All authors have conducted research on parental mental health and child outcomes. CL has extensive professional experience working with and providing perinatal services to young parents in deprived areas. Three authors (ER, CL and LJD) have experience of becoming and being a parent in the UK.

The study was conducted in the UK during the COVID-19 pandemic from February 2021 to May 2021. In the UK the first lockdown was announced on 23^rd^ March 2020 and lasted until the beginning of June 2020. A second and third lockdown lasted from November 2020 until March 2021.

### Ethical considerations

Participants were provided with local and national support contacts prior to taking part. Participants were given the option to complete the interview on the telephone or online and had the option to speak to either a female or male interviewer. A safeguarding protocol was in place but was not triggered at any time during the study. No participant withdrew from the study and no significant distress was reported in response to the interview process. Participants were reminded that they could stop the interview at any time without giving a reason. The research team abstained from asking participants specific questions about their ethnicity and socio-economic status to minimise risks of potential distress and disengagement. Identifying information was removed from transcripts to protect participants’ anonymity. The study received ethical approval from the Research Ethics Committees of the University of Oxford (R72496) and the National Society for the Prevention of Cruelty to Children (R.20.193). All methods were performed in accordance with the relevant guidelines and regulations.

## Results

### Participants

Twenty-one parents (6 male, 15 female) were included in the study; this included two sets of parents who were in a relationship with one another. Participants’ mean age was 22.04 years, including one slightly older father (27 years) who at the time of the interview was in a relationship with a young mother who met the study’s eligibility criteria. Participant characteristics are presented in Table 2.

**Table 2:** Characteristics of parents interviewed

Variables	*N*
**Age**	
16–20 years	5
21–22 years	4
23–24 years	11
> 24 years	1
**Living situation**	
Alone	2
With child(ren) or partner	5^c^
With partner and child(ren)	11
With family/parents and child(ren)	3
**Relationship with partner**	
In relationship with partner	14
Separated from partner	7
**Number of children at time of interview**	
Pregnant with 1^st^ child and/or has one child	11
More than one child and/or pregnant with next child	10
**Parenting status**^**b**^	
First time parent during COVID-19 pandemic	11
Pregnant at time of interview	4
Pregnant during COVID-19 pandemic^a^	16
Gave birth during COVID-19 pandemic^a^	11
Postnatal during COVID-19 pandemic^a^	19

^a^‘during COVID-19 pandemic’ refers to time period after March 2019, ^b^some parents are included in multiple categories: e.g., pregnancy and birth or birth of one child and pregnancy with second child during the pandemic. ^c^these categories were combined, due to small cell count, to protect participants’ identity

Most parents (term is used to include both mothers and fathers) reported that the pandemic created a range of challenges in relation to becoming and being a parent. Alongside this, parents described struggling with their mental health and wellbeing. Overall, three themes and seven sub-themes were identified as shown in Table 3 below.

**Table 3:** Overview of identified themes

Primary theme	Sub-themes
1. COVID-19 specific anxieties and stressors	1.1 Consequences of contracting COVID-19
	1.2 High levels of uncertainty
2. Loneliness, isolation and lack of social support	2.1 Navigating pregnancy alone
	2.2 Parenting in isolation
3. Disruptions to perinatal care	3.1 Availability of professional support and care
	3.2 Communicating with professionals
	3.3 Phone, online and alternative support from professionals

#### Theme 1: COVID-19 specific anxieties and stressors

Parents described the period of becoming and being a parent during the pandemic as stressful and anxiety provoking. This is discussed under two sub-themes. The first sub-theme focuses on parents’ reports around specific anxieties of contracting the virus and the possible consequences. The second theme explores parents’ concerns regarding the high levels of uncertainty throughout the pandemic.

##### Sub-theme 1: Consequences of contracting COVID-19

*“Because of COVID I don’t know whether to send her to nursery or not. I don’t know whether that’s putting my new-born at more risk. It’s really a difficult time and I don’t have anyone to ask really.”*

(Participant 5, mother, aged 20 years).

Parents stated that their anxiety of contracting coronavirus was further amplified by comments and recommendations from friends, families and professionals to ‘*stay at home and not leave the house*.’ There were some parents who reported that they had not left the house for months, which they described had had a significant impact on their mental health and wellbeing.

“*They said if your partner catches Coronavirus, then she could potentially lose her baby*. […] *I’ve been stuck indoors, not going out, making sure that the baby’s health is unaffected as a result […] my mum also said, don’t go out at all, so, I didn’t go out since July, August, September, just before Christmas, I didn’t go out at all, I only went out when necessary*.”

(Participant 4, father, aged 24 years)

Parents were often worried about not being able to care or provide for their family if they contracted COVID-19. Mothers’ concerns appeared to centre on the risk of becoming severely ill and not being able to physically care for their children, particularly due to the absence of practical help from others outside their household during lockdown. Fathers primarily reported worries about possible and actual financial consequence of not being able to work due to illness or quarantine rules.

*“I was no longer in work at the time. I had to claim benefits. So, as a result, I struggled to put food on the table, which I felt a lot of guilt, a lot of shame…that is why we suffered”*

(Participant 4, father, aged 24 years)

##### Sub-theme 2: High levels of uncertainty

Parents often described multiple changes across different areas of their lives arising from the pandemic and associated restrictions, which created considerable feelings of uncertainty. For example, some parents were unsure about the security of their job or furlough payments resulting in financial concerns, while others were unsure about the implications of COVID-19 guidelines for their perinatal appointments, birth plan or support from professionals or family members. Parents described their perception that the dynamic nature of COVID-19 restrictions further contributed to their ability to adjust to the new ways of living.

“*It was a bit – everything was all over the place and it was a big change which was hard to obviously try and change everything at once, and then it was like you got used to one change and then something changed, and then it was like this second time around, whatever was said on the first time doesn’t matter because [everything changed again*]”

(Participant 8, mother, aged 22 years)

Expectant parents reported being unsure about the changing hospital guidelines, and worried how this might affect their maternity care, for instance if the birth-plan they had prepared would still be followed. Additionally, they reported being worried about the uncertainty of accessing items to prepare for their baby’s arrival, including clothing, equipment or bedding. Parents with a new-born baby described it as challenging to access food and baby supplies such as formula milk and nappies during the pandemic.

*“I had to go on to formula but it was a proper struggle trying to find a formula, you’d have to go to probably about 10 shops before you found it. So, I pretty much had everyone I knew out looking for it, so that was stressful, because there was nothing else I could give her.”*

(Participant 13, mother, aged 22 years)

#### Theme 2: Loneliness, isolation and lack of social support

Young parents described the first year of the pandemic as a lonely and isolating experience. The legal limit on social contact was perceived as one of the greatest challenges. Parents attributed their feelings of depression, helplessness and hopelessness to their experience of persistent loneliness and lack of social support. These issues are discussed in two sub-themes: (1) navigating pregnancy alone and (2) parenting in isolation.

##### Sub-theme 1: Navigating pregnancy alone

Parents stated that COVID-19 restrictions across healthcare settings necessitated many expectant mothers to attend maternity care appointments on their own which they experienced as stressful: ‘*Having to go alone was quite scary […] at the beginning I wanted to cry every time I walked into the hospital*.’ This was particularly challenging for mothers who experienced complications during their pregnancy. On occasion, mothers reported that they had wanted exceptions to be made when complications occurred, or when they had to attend additional appointments for further check-ups or procedures.

*“I think because I’m doing it on my own and I get worked up, and I get stressed out,– I’m looking at the scan screen and I keep looking back and forth and I think that worries me more, because he [the father] is not there to give me that support and he’s not holding my hand. […] It was the waiting on my own to get the test done and everything,[…], and then having to come home and tell him [the father] about the extra fluid, it literally got me to the point where I broke down before I even told him I was that worked up about it.”*

(Participant 16, mother, aged 24 years)

Mothers emphasised the additional burden of taking sole responsibility during appointments when they were asked to make medical decisions without being able to consult their partners (or another significant adult in their life). Communicating important medical information to partners after the appointment without professional support was reported as being particularly difficult for some mothers. More often, mothers described feeling overwhelmed trying to answer questions from their partner or family members, reporting that they often could not remember, or had not fully understood the information given by the healthcare team during the appointment. Mothers found it emotionally challenging when they had to share *‘bad news’* with the baby’s father after the appointment, such as complications with the baby’s development or pregnancy. Mothers believed it would have been helpful if at least one other person could have been present at the appointment, even if this was not the father, especially when complications arose or were expected.

“*It was just all on my shoulders, like, going to the appointments and things. Whereas, a problem shared is a problem halved. Whereas if there was an issue at any of the scans it would have been mine and my partner’s problem, not just mine.*”

(Participant 11, mother, aged 23 years)

“*I would have to hear the bad news with this baby, and then go home and have to tell my partner the bad news again. If we were there together it would have just been one lot of bad news whereas if I have to repeat it, it’s – it gets a lot.” […] He had a lot of questions and I just didn’t really have the answers to them.” *

(Participant 7, mother, aged 20 years)

Parents described feeling guilty when fathers were ‘*missing out’* on key moments during the pregnancy such as hearing the baby’s heartbeat. Often, fathers felt that not being able to attend antenatal appointments meant they had a lack of understanding about the pregnancy process. Parents felt as if fathers had been ‘*pushed out*’ by the regulations and as a result some mothers feared that that their partners were less engaged with the pregnancy and worried what impact that would have after the baby was born.

“*I was really hoping for him to be more connected with the pregnancy, but he wasn’t really, because he wasn’t really involved. He couldn’t even come to the Midwife appointments […] with my first pregnancy, he’d be a lot more involved. […] my partner used to swap his shift to come to appointments, but it’s only a phone call appointment, you know, there’s no point him swapping his shift for that kind of thing.*”

(Participant 15, mother, aged 18 years)

Mothers also described their perceptions of the negative effects that not attending appointments had on their partner’s mental health, especially when there were difficulties during the pregnancy or with the baby.

“W*hen they couldn’t find the heartbeat and stuff like that, it was important that he was there, but he couldn’t be, so that was hard in itself because that then made his depression worse because he was then thinking something is wrong with the baby, and everything.”*

(Participant 9, mother, aged 22 years)

Most fathers described not being able to be at appointments as highly stressful; they expressed a strong desire to be with their partner and child during appointments, especially if the mother had to stay in hospital for several days. Fathers felt it would have been helpful if appointments could have been recorded or if they could have connected virtually to the appointment; this was not reported to have happened or been offered.

*“Very, very- like a very anxious situation. It was- was like I’d always stand outside the hospital. But I’d never put my phone away. Just sat there hoping that like if anything went wrong, she’d get the contact- find a way of contacting me somehow. But yes, there was definitely a lot more anxiety with the hospital scans. Because I’d still be there. I’d still take her to every appointment but I’d just be stood outside*.”

(Participant 19, father, aged 24 years)

*“My partner wasn’t allowed her phone on, it would have been nice if they would have been able to be like “Do you want to video it” so that they can record it, so even if it was like FaceTime or something but there were none of those options.”*

(Participant 10, father, aged 27 years)

##### Sub-theme 2: Parenting in isolation

Parents with children reported that they missed having access to any type of in-person support and a social network. This included situations of getting a ‘*helping hand’* from a family member or friend, but also being able to join local parent groups.

***“****I became isolated in my home, I already suffered with post-natal depression, after having my son. So, when COVID hit, it just got worse I couldn’t get any support from anywhere, I was a new mum, first time mum as well. So, I had no experience of being a mum, I had no idea what I was supposed to do, am I doing something bad, am I doing something wrong; are other mums feeling like I’m feeling, are their kids doing what my kid’s doing? So, when COVID happened, I couldn’t experience it with any mums, I couldn’t see my family to get advice from*.”

(Participant 2, mother, aged 22 years)

Parents who had been able to attend local parenting support groups pre-pandemic reflected on the benefits of being able to share their experiences with other parents and asking questions about how to care for their babies. They felt this had provided them with comfort and eased feelings of insecurity about being a new parent. One mother reflected that attending parent groups prior to the pandemic had helped her overcome the stigma she felt being a young parent, as well as finding her role as a new mother and feeling more confident.

“*The rest are like same age as me and they’re not, they’re not having kids so I think it was quite nice to meet those people [other parents] even though they are older than me […]. I think it helps me feel more confident in my choices. Like my friends have helped me because, they’re friends that I met at baby group. They both have children before so I think they really helped me with setting routines and thinking about weening and stuff like that because I had no idea.”*

(Participant 12, mother, aged 23 years).

Parents described not being able to have family or friends present to help with the practical aspects of parenting due to COVID-19 restrictions as challenging. This was especially evident for mothers who lived without a partner. Some parents reported having to ‘*break the rules’* by visiting a family member, often their own parents, as a way to manage their parenting tasks but also to help with continuous social isolation. However, this was accompanied by strong feelings of guilt and anxiety.

*“I was just really not coping well at all and I just kind of thought for the sake of myself I need to get out of this house. That was probably the best thing I did. Also horrible because I just felt like the police were coming after me all the time.”*

(Participant 12, mother, aged 23 years).

The majority of young parents suggested that having at least one other adult contact (especially in the first couple of weeks after giving birth) and opportunities to attend ‘*socially distanced’* or ‘*online group*s’, would have been beneficial.

*“Just making sure that somebody has at least one concrete support network that they can turn to […] just somebody to speak to because it does get difficult.”*

(Participant 3, mother, aged 23 years)

#### Theme 3: Disruptions to perinatal care

Parents perceived that the pandemic and the associated restrictions had a significant impact on perinatal services. Perinatal services refer to both informal and formal health and social care services provided to parents in the UK during and post pregnancy by the national health service (NHS), local authorities, third sector organisations and community or local volunteer groups. Parents reported various changes to perinatal services including temporary closure of community centres, relocation of services and moving in-person care to online. Parents’ perceived impact of these disruptions are discussed in three sub-themes: (1) availability professional support and care, (2) communicating with professionals and (3) phone, online and alternative support from professionals.

##### Sub-theme 1 Availability of professional support and care

Parents thought there may have been a delay in the provision of perinatal care especially at the beginning of the pandemic, with many describing feeling *‘as if they had been forgotten about.’* Often, parents stated they were confused about how and who to contact if they needed help with their own or child’s health.

*“It probably would have been nice to know that like we were thought about. It just felt like we were just, it probably felt like that for everyone but it just felt like we were all forgotten like just stay at home, don’t be seen you know. […] It was just like they’d vanished. I mean still now, over a year later, and I’ve still not heard anything.”*

(Participant 8, mother, aged 22 years)

Some parents reported that services had improved after restrictions eased. Other mothers described feeling *‘well supported’* throughout the pandemic by their health visitor, midwife or family nurse, who had continued to emphasise their accessibility and *‘could be called anytime’*.

*“She actually was in touch with me throughout the whole pregnancy. I mean she’s very supportive. I literally take my hat off to her, she’s always been there for me*.”

(Participant 9, mother, aged 22 years)

Parents highlighted the important role played by community centres and health visitors pre-pandemic; they described these as their ‘*one stop shop’* or main access point to seek advice and information regarding parenting. Expectant parents stated that they felt less prepared than they would have liked for their baby’s arrival, which they attributed to being unable to attend antenatal classes. Parents with new-born babies emphasised the absence of social and practical support which they had previously received from community centres; this included accessing vitamin prescriptions, food parcels, clothes or toys for their baby. There were also parents who expressed their gratitude about the efforts their local community centre had made to support them, such as delivering parcels of clothes and toys.

Parents indicated that prior to the pandemic health visitors, family nurses and community centres had been vital in sign posting them to relevant support services. Changes in parents’ ability to access these services during the pandemic meant that many felt ‘*lost’* as they were unsure how to access support.

*“It was the social side of things and getting advice. Obviously, you could access health visitors, and there was all sorts of services, like stop smoking, you could get food parcels, everything pretty much in need would be under one roof and obviously that’s no longer available now.”*

(Participant 8, mother, aged 22 years)

Parents stated they were concerned about the impact of suspended face to face services on their baby’s physical, social and emotional development. Many parents had previously accessed community services including parent-baby groups, and felt that without these services they were lacking both the resources (e.g. space, toys and books) and the knowledge on how they could foster their child’s development.

*“It’s just a bit difficult because with a baby, it’s all about trying to stimulate the development, stimulate the brain and things like that. But we couldn’t attend any baby sensory classes, we couldn’t just go and speak to the health visitor or have the health visitor visit. We can’t go to the sessions and things like that because everywhere is closed. So, it’s a bit rubbish, he doesn’t get the social development side.”*

(Participant 3, mother, aged 23 years)

Concerns about their own or their partner’s mental health were raised by almost all parents. Many described experiencing an exacerbation in their feelings of anxiety and low mood during the pandemic, with some engaging in self-harming behaviours. These parents reported that they had wanted help for these psychological issues during the pandemic, but many experienced difficulties or delays in accessing mental health support: ‘*nobody got involved, I didn’t have anyone from the mental health team get involved until my son was 7 months old.’* For parents who had received mental health support during the pandemic, they perceived the process as *‘long and difficult’*, with the support being offered as ‘*too late’*; for instance, only obtaining help after a hospital Emergency Department attendance for a ‘*mental health crisis*.’ Parents felt that in some cases the delayed or lack of support had contributed to a deterioration in their mental health, which they believed could have been prevented by earlier support.

*“Honestly, I’m at that point I don’t know what to do. I don’t know how to help him. I can’t even. I don’t know what to do about that. How do I get him to the point where he can see a Counsellor during these times.”*

(Participant 5, mother, aged 20 years)

Other mothers also highlighted the important role of health visitors to proactively identify parents at risk of mental health difficulties. Parents explained that they did not realise that they were struggling because ‘*you don’t know when you’re a first-time mum’, or ‘felt too low in mood to ask for help’* and ended up ‘*suffering in silence*’.

Parents felt that the decline in their mental health also affected their abilities to be a supportive partner and parent. Both mothers and fathers described a lack of support for fathers’ mental health and parenting, which they perceived as ‘*unfair’*.

“*The depression then affected his ability to be a dad because he then wasn’t spending as much time as he wants to with his son because he was not getting the help that he needed to be able to do that*. *[…]* *dads are not offered half as much support and it really does – if one of you is not okay then both of you are not going to be that okay.*.”

(Participant 5, mother, aged 20)

##### Sub-theme 2: Communicating with professionals

Parents expressed a desire for better communication with perinatal services, including information and regular updates about a) current COVID-19 restrictions b) which and how services had changed c) how parents could access services during the pandemic and d) updates for fathers about mother and child when they were unable to attend appointments.

Parents reported that they had been unsuccessful in contacting different professionals, including social services, the council, their GP, midwife or health visitor and charities in order to get support, and felt that they were often ‘*going around in circles*’. Some parents felt that they could not find sufficient information on how to contact different services.

“I felt a bit **** [bad] as a parent. It was little things that wouldn’t normally matter but that’s because it was always accessible. I could just pick up the phone, she’d [health visitor] answer. I’d ring her mobile. Or I could ring the local board of health visitors and someone would get back in touch. But there’s just nothing. It was like everyone has disappeared. [... ] I mean I got more help asking Argos, online chat, how to place an order or return an order during the pandemic than I did on contacting about my child’s health.”

(Participant 8, mother, aged 22)

Mothers reported that they were unsure whether organisations had been offering alternative provision, and were therefore potentially not accessing services that might have been available.

*“No, we didn’t do any of the online stuff. Partly because I’ve never heard of any in [place]. I didn’t hear of any online groups.”*

(Participant 15, mother, aged 18)

Parents wanted more clarity and specific explanations from professionals around COVID-19 regulations and restrictions. Many described feeling confused about the rationale for some regulations: ‘*I just didn’t see what the difference was with taking your partner at 12 weeks but not 20 weeks.’*

When mothers experienced complications or were hospitalised, fathers reported feeling stressed and concerned at the absence of updates (in some cases for days) from the medical staff about the mother or child’s condition.

*“Very stressful because obviously not all the time she was able to let me know how things are going because she’s sleeping and all of this. She was quite exhausted from it all. So there was one point where she text me, she goes, ‘They’re going to break my waters’, then I’ve called up the hospital and said, like what’s happening? And they basically said that they’re not planning on doing anything. So it’s sort of, I got put in a position where I didn’t know what was going on. And then I’m panicking because I feel like I’m going to miss the birth”*

(Participant 21, father aged 16 years)

##### Sub-theme 3: Phone, online and alternative support from professionals

Parents believed that many organisations attempted to replicate normal services during lockdown by using the phone or online platforms. Many parents acknowledged the efforts of organisations to provide this remote care, but there were also parents who reported having significant difficulties with accessing digital services; some had no internet connection or could not afford the amount of data needed to conduct video calls, which occasionally was intensified by additional financial strains due to the pandemic.

*“One of the issues is obviously a lot of people don’t have internet and at the time people are facing massive financial difficulties [...]. Why couldn’t they print off a questionnaire and put it to all the parents that were in contact, and you could have responded, give them a pre-paid envelope, and post it back, but it didn’t happen.”*

(Participant 8, mother, aged 22 years)

The transition to tele- or digital care provision was perceived as effecting the support available to parents during the pandemic. For instance, some mothers described feeling worried about their child’s development, and wanted professionals to be able to observe their child in-person to identify any problems and to make necessary onward referrals.

*“She asked me all these questions and I kind of just didn’t know the answer or he wasn’t doing it and then that kind of made me feel like he was not progressing properly. It just doesn’t work over the phone like they need to see how he’s behaving. And make sure everything is basically fine and normal, because you’re on the phone it gets a bit worrying.”*

(Participant 12, mother, aged 23 years)

The lack of in-person support was particularly challenging for first-time parents, mothers experiencing difficulties with breastfeeding and mothers needing treatment for birth-related injuries. In some situations, online and phone consultations were perceived as obstacles for seeking help by parents and were considered unhelpful, because parents felt uncomfortable showing certain aspects of their life or baby on a video call. Some parents said that they needed more ‘*hands-on’* support, for example, so that the health visitor or midwife could demonstrate how to hold the baby or check their breastfeeding technique. Similarly, many parents highlighted the potential room for improvement in the leaflets and video materials provided, as they felt that the information was insufficiently engaging or too generic to help with their specific problem.

*“When I spoke to the midwife and the health visitor about it they literally just gave me leaflets and a video to watch on YouTube about breastfeeding. (…) I don’t think it really helped, and I don’t think it would help new parents because it didn’t give that much information. The leaflets showed you like little diagrams but didn’t actually show you how to hold the baby or how to get him to latch on or anything.”*

(Participant 16, mother, aged 24 years)

*“You could also just Google anything and Google would tell me an answer if I wanted to, but I really wanted to speak to the person that knew my child, and that knew what she was like and they would know what was best for her, not for an average child. And that’s obviously what they’re there for.”*

(Participant 8, mother, aged 22 years)

Some parents who described struggling with their mental health reported parent groups on social media or specific parent apps very helpful ‘*I relied on that group massively’* and ‘*it was the biggest go to’*. However, others also shared their concerns about the validity of some of the advice given by other users.

A few parents did access online therapy during the pandemic, but they reported having difficulties with this metho. These included struggling to ‘*open-up*’ emotionally over the phone, as well as the practical challenges of being at home and/or having to look after their children. One mother shared her distress about losing her therapy slot, because she had been unable to answer the phone in time.

*“That was probably one of the things where COVID has most affected one of my appointments because had I have been at a physical appointment, I wouldn’t have missed it… it wasn’t intentional for me to miss it, like a phone call only goes off for, what is it? Like 30 seconds and if I wasn’t at my phone at that exact minute, I can’t help that. And also, with it being a no-caller ID, I couldn’t even call back and they only tried to call once.”*

(Participant 15, mother, aged 18 years)

## Discussion

Parents in this study described numerous challenges during the pandemic, including high levels of anxiety, continuing uncertainty, loneliness and isolation, and not knowing where to get help from when it was needed. Consequently, parents felt that COVID-19 had adversely impacted their parenting, mental health and wellbeing.

Parents’ anxiety about contracting COVID-19 was exacerbated by the perceived lack of information available throughout the pandemic. While there has been a rapid increase in the scientific understanding about the effects of the virus since March 2020, evidence about the impact of coronavirus on mothers and babies has only recently been published (https://www.rcog.org.uk/en/guidelines-research-services/coronavirus-covid-19-pregnancy-and-womens-health/). Healthcare professionals can play a key role in updating parents with the latest scientific guidance; a recent study demonstrated that pregnant women who had been given information about the potential effects of COVID-19 on pregnancy from a healthcare professional had significantly lower levels of anxiety and depression [30].

Many parents in this study described a range of issues which made it more challenging to engage with aspects of perinatal care. This is particularly important to address in the context of previous research highlighting the ‘unique healthcare needs’ [31] of adolescent parents; for example, adolescent mothers report increased pain, fear and lack of control during labour [32] and less satisfaction with inpatient postpartum nursing care (relative to married adult mothers) [33].The importance of including fathers in their baby’s antenatal care when physical attendance at appointments is not possible should be considered a priority if future restrictions are required. Mothers in this study described feeling overwhelmed by the responsibility of attending appointments alone, while fathers felt excluded and stressed about the absence of information given to them about their partner’s or child’s health. This could be addressed by facilitating mothers to record their baby’s heartbeat and, where possible, video calls could be used. COVID-19 restrictions on fathers’ attendance at appointments may impact on their understanding of complications during the pregnancy. This could be mitigated by ensuring that parents have a named professional who acts as a point of contact after appointments to answer questions. However, this has workforce implications and may not always be possible due to the multiple demands on healthcare services during a pandemic.

For many young people, pregnancy and parenting are major risk factors for mental health difficulties, which may have implications for their child’s wellbeing [34]. Informal and professional support networks for young people have been disrupted by the pandemic. Health visitors provide support to parents and children across a wide range of areas including development, physical and emotional wellbeing. However, over recent years changes in funding have resulted in a 31% reduction in the number of health visitors [35]. Furthermore, during the pandemic, health and social care professionals’ experienced increased caseloads, had limited access to personal protective equipment (PPE) and many (up to 50% in certain areas) were re-deployed to other healthcare sectors [35]. Health visitors have reported their concerns about the impact of the pandemic on parents and their children, with recent reports indicating an 81% increase in perinatal mental illness and 61% increase in child neglect [35, 36]. Many parents in this study highlighted needs which had not been met, for example relating to their own or a partner’s mental health, or their child’s health and development. It is essential that services do not overlook the legacy of the pandemic for young parents and offer timely and appropriate support to address outstanding or ongoing concerns. The well-documented implications of the first 1001 days for children’s long-term outcomes illustrates the importance of providing urgent support to families for whom this critical period occurred during the pandemic.

Technology played an increasingly important role for parents during the pandemic. While some parents in this study highlighted obstacles for accessing online support such as internet and mobile data charges, many reported successfully using online platforms and social media groups for help. Research exploring the acceptability and effectiveness of technology-based interventions for parents, (e.g., O’Mahen et al., [37]) suggests that they represent promising means to supporting parents in the perinatal period. Nevertheless, barriers due to internet poverty must be urgently addressed to ensure that vulnerable groups are not excluded by a shift towards online care provision.

### Strengths and limitations

To the researchers’ knowledge, this is the first study that specifically reports both young mothers’ and fathers’ experiences during the COVID-19 pandemic. The study could be strengthened by addressing the participant gender imbalance and triangulating the experiences of young parents with the perspectives of members of their extended families and healthcare staff. Recent evidence suggests that different ethnic and racial groups from deprived areas are at an increased risk of experiencing mental and physical ill-health during the pandemic [2, 3].Future research should specifically include parents from diverse racial and ethnic communities to elucidate their experiences and needs. Due to the present convenience sampling approach, the findings may be subject to a sampling bias, whereby certain participants may have been more likely to be included in the present study [38] or motivated by the financial incentive for participation. Although parents in this study reported significant changes in perinatal services, it is unclear to which extent these findings are generalisable across the UK.

## Conclusion

This study highlights the significant impact of the COVID-19 pandemic on young parents, especially in terms of their mental health and parenting. Parents’ psychological distress during the pandemic was exacerbated by changes in service provision and delivery, leaving many parents feeling lonely, helpless and overwhelmed.

The insights and experiences of young parents shared in this study are of great importance for policy and practice; specifically (a) what services are needed to mitigate the impact of the COVID-19 pandemic and (b) how challenges for parents and provision of care could be addressed if further restrictions are necessary in this, or future pandemics. Recommendations based on this research study include: (a) facilitating and supporting the involvement of both parents during perinatal appointments; (b) effective identification and provision of early parental mental health support; (c) improving communication pathways between parents and professionals to reduce levels of uncertainty and clarify sources of help, and (d) ensuring equity in accessing online services.

## Data Availability

The datasets generated and/or analysed during the current study are not publicly available to protect participants, coding frames and analysis steps are available from the corresponding author on reasonable request.

## References

[1] Department of Health and Social Care, “COVID-19 mental health and wellbeing recovery action plan,” 2021. [Online]. Available: https://www.gov.uk/government/publications/covid-19-mental-health-and-wellbeing-recovery-action-plan/covid-19-mental-health-and-wellbeing-recovery-action-plan#fn:1. [Accessed: 02-Sep-2021].

[2] Pierce M, et al. Mental health before and during the COVID-19 pandemic: a longitudinal probability sample survey of the UK population. Lancet Psychiatry. 2020;7(10):883–92. 10.1016/S2215-0366(20)30308-4 PMC7373389 32707037

[3] Fortuna LR, Tolou-Shams M, Robles-Ramamurthy B, Porche MV (2020). Inequity and the Disproportionate Impact of COVID-19 on Communities of Color in the United States: The Need for a Trauma-Informed Social Justice Response. Psychol Trauma Theory Res Pract Policy.

[4] Hodgkinson S, Beers L, Southammakosane C, Lewin A (2014). Addressing the mental health needs of pregnant and parenting adolescents. Pediatrics.

[5] Malabarey J, Balayla SL, Klam S, Shrim A and Abenhaim H. Pregnancies in young adolescent mothers: a population-based study on 37 million births. J Pediatr Adolesc Gynecol. 2012;25:98–102. 10.1016/j.jpag.2011.09.004 22088316

[6] Lipman EL, Georgiades K, Boyle MH (2011). Young adult outcomes of children born to teen mothers: Effects of being born during their teen or later years. J Am Acad Child Adolesc Psychiatry.

[7] Wachs TD, Black MM, Engle PL (2009). Maternal depression: A global threat to children’s health, development, and behavior and to human rights. Child Dev Perspect.

[8] Fegert JM, Vitiello B, Plener PL, Clemens V (2020). Challenges and burden of the Coronavirus 2019 (COVID-19) pandemic for child and adolescent mental health: A narrative review to highlight clinical and research needs in the acute phase and the long return to normality. Child Adolesc Psychiatry Ment Health.

[9] Matsushima M and Horiguchi H. The COVID-19 pandemic and mental well-being of pregnant women in Japan: Need for Economic and Social Policy interventions. Disaster Med. Public Health Prep. 2020. 10.1017/dmp.2020.334. 10.1017/dmp.2020.334 PMC7642494 32907687

[10] Fan S (2020). Psychological effects caused by COVID-19 pandemic on pregnant women: A systematic review with meta-analysis. Asian J Psychiatr.

[11] Suwalska J, et al. Perinatal Mental Health during COVID-19 Pandemic : An Integrative Review and Implications for Clinical Practice. J Clin Med. 2021;10:2406. 10.3390/jcm10112406 PMC8199229 34072357

[12] Kinser PA (2021). Depression, Anxiety, Resilience, and Coping: The Experience of Pregnant and New Mothers during the First Few Months of the COVID-19 Pandemic. J Women’s Heal.

[13] Sweet L (2021). Becoming a mother in the ‘new’ social world in Australia during the first wave of the COVID-19 pandemic. Midwifery.

[14] Vasilevski V et al*. *Receiving maternity care during the COVID-19 pandemic: Experiences of women’s partners and support persons. Women Birth. 2021. 10.1016/j.wombi.2021.04.012. 10.1016/j.wombi.2021.04.012 PMC8075817 33941497

[15] Yan H, Ding Y, Guo W (2020). Mental Health of Pregnant and Postpartum Women During the Coronavirus Disease 2019 Pandemic: A Systematic Review and Meta-Analysis. Front Psychol.

[16] Hessami K, Romanelli C, Chiurazzi M, Cozzolino M (2020). COVID-19 pandemic and maternal mental health: a systematic review and meta-analysis. J Matern Neonatal Med.

[17] Stein A (2014). Effects of perinatal mental disorders on the fetus and child. Lancet.

[18] Thomas E (2021). Covid: Being alone in pregnancy due to hospital rules.

[19] Baldwin S (2020). Working with fathers: Reflections and learning from the New Dad Study. J Heal Visit.

[20] Menzies J. Forgotten fathers : The impact appropriate and timely information. J Heal Visit. 2021;9(4):150–3.

[21] Recto P, Lesser J (2021). Young Hispanic fathers during COVID-19: Balancing parenthood, finding strength, and maintaining hope. Public Health Nurs.

[22] Leach LS, Poyser C, Cooklin AR, Giallo R (2016). Prevalence and course of anxiety disorders (and symptom levels) in men across the perinatal period: A systematic review. J Affect Disord.

[23] Giallo R (2012). Father mental health during the early parenting period: Results of an Australian population based longitudinal study. Soc Psychiatry Psychiatr Epidemiol.

[24] Baldwin S, Malone M, Sandall J, Bick D (2018). Mental health and wellbeing during the transition to fatherhood: A systematic review of first time fathers’ experiences. JBI Database Syst Rev Implement Reports.

[25] Ramchandani PG, Stein A, O’Connor TG, Heron J, Murray L, Evans J (2008). Depression in men in the postnatal period and later child psychopathology: A population cohort study. J Am Acad Child Adolesc Psychiatry.

[26] O’Brien BC, Harris IB, Beckman TJ, Reed DA, Cook DA (2014). Standards for reporting qualitative research: A synthesis of recommendations. Acad Med.

[27] Braun V, Clarke V (2006). Qualitative Research in Psychology Using thematic analysis in psychology Using thematic analysis in psychology. Qual Res Psychol.

[28] Braun V, Clarke V (2021). Can I use TA? Should I use TA? Should I not use TA? Comparing reflexive thematic analysis and other pattern-based qualitative analytic approaches. Couns Psychother Res.

[29] Braun V, Clarke V (2020). One size fits all? What counts as quality practice in (reflexive) thematic analysis?. Qual Res Psychol.

[30] KahyaogluSut H, Kucukkaya B (2021). Anxiety, depression, and related factors in pregnant women during the COVID-19 pandemic in Turkey: A web-based cross-sectional study. Perspect Psychiatr Care.

[31] Harrison ME, Clarkin C, Rohde K, Worth K, Fleming N (2017). Treat Me But Don’t Judge Me: A Qualitative Examination of Health Care Experiences of Pregnant and Parenting Youth. J Pediatr Adolesc Gynecol.

[32] Zasloff E, Schytt E, Waldenstrom U (2007). First time mothers’ pregnancy and birth experiences varying by age. Acta Obstet Gynecol Scand.

[33] Peterson WE, DiCenso A (2002). A Comparison of Adolescent and Adult Mothers’ Satisfaction with Their Postpartum Nursing Care. Can J Nurs Res.

[34] Siegel RS, Brandon AR (2014). Adolescents, pregnancy, and mental health. J Pediatr Adolesc Gynecol.

[35] Institute of Health Visiting. State of Health Visiting Survey Reports: State of health visiting in England. 2020. State-of-Health-Visiting-survey-2020-FINAL-VERSION-18.12.20.pdf (ihv.org.uk). Accessed 2 Sept 2021.

[36] Conti G, Dow A (2020). The impacts of COVID-19 on Health Visiting in England.

[37] O’Mahen HA (2014). Netmums: a phase II randomized controlled trial of a guided Internet behavioural activation treatment for postpartum depression. Psychol Med.

[38] Hedt BL, Pagano M (2011). Health indicators: Eliminating bias from convenience sampling estimators. Stat Med.

